# Role of the Medial Agranular Cortex in Sensory-Motor Integration During Walking: Insights From a Rodent Stroke Model

**DOI:** 10.7759/cureus.96737

**Published:** 2025-11-13

**Authors:** Daisuke Ishii, Hironobu Osaki, Arito Yozu, Tatsuya Yamamoto, Satoshi Yamamoto, Mariko Miyata, Yutaka Kohno

**Affiliations:** 1 Department of Cognitive and Behavioral Neuroscience, Graduate School of Biomedical and Health Sciences, Hiroshima University, Hiroshima, JPN; 2 Laboratory of Functional Brain Circuit Construction, Graduate School of Brain Science, Doshisha University, Kyoto, JPN; 3 Department of Rehabilitation Medicine, Nippon Medical School, Tokyo, JPN; 4 Faculty of Medical and Health Sciences, Tsukuba International University, Ibaraki, JPN; 5 Department of Physical Therapy, Ibaraki Prefectural University of Health Sciences, Ibaraki, JPN; 6 Division of Neurophysiology, Department of Physiology, School of Medicine, Tokyo Women’s Medical University, Tokyo, JPN; 7 Center for Medical Sciences, Ibaraki Prefectural University of Health Sciences, Ibaraki, JPN

**Keywords:** forelimb, hindlimb, motor cortex, stroke, walking

## Abstract

In the rodent motor cortex, limb movements are primarily governed by the lateral agranular cortex (AGl), whereas the medial agranular cortex (AGm) has been proposed to contribute to higher-order aspects of motor control. However, its independent contribution to sensory-motor integration has not been established. Previous studies have shown that infarcts encompassing both the AGl and AGm cause contralateral motor impairments, suggesting, but not proving, that the AGm might participate in movement control. In this study, we specifically tested whether focal lesions restricted to the AGm impair skilled locomotion. Using the ladder rung walking task, which requires precise paw placement based primarily on somatosensory cues with additional visual contributions, we examined mice with focal photothrombotic infarcts centered within the AGm at +2.0, +1.5, +1.0, or +0.5 mm anterior to bregma. To enable objective assessment, we developed an automated analysis pipeline using DeepLabCut^TM^ (v2.2.0.4), a markerless pose-detection machine learning Python package, that computed forelimb and hindlimb foot-fault rates from multi-view videos. Foot-fault rates for both limbs did not increase under either regular or irregular rung patterns across pre- and postoperative sessions. By isolating the functional contribution of the AGm from that of the AGl, our results refine previous interpretations and demonstrate that AGm lesions alone do not impair visually and somatosensorily guided limb movements. Within the sensitivity of this assay, these findings indicate that the AGm is not required for executing such movements on this task. Together with prior evidence, these findings support the view that the AGm contributes primarily to the higher-order modulation of motor behavior, likely through spatial attention, rather than to direct motor execution.

## Introduction

In rodents, the medial agranular cortex (AGm) is situated in the medial forebrain and has been linked to spatial attention [1-3]. The AGm links sensory cues to motor actions and serves as a key node in the circuitry regulating voluntary behavior [4]. Anatomically, the AGm is considered homologous to the frontal eye fields, arcuate cortex, or area 8 in primates [5,6]. Convergent inputs arise from the ipsilateral thalamus, the contralateral AGm, and the ipsilateral visual, somatosensory, and auditory cortices, as well as the posterior parietal and lateral orbital cortices [6]. These inputs provide AGm with multimodal sensory information necessary for context-dependent motor planning.

The AGm contains corticospinal projection neurons and maintains reciprocal long-range connections with S1, where top-down AGm input drives layer 5 activity and disruption of this pathway degrades tactile perception [7,8]. Lesion studies in rats have suggested that the AGm subserves functions comparable to those of the premotor cortex in primates, particularly in coordinating sensory inputs with motor outputs [9,10]. Based on these anatomical and physiological properties, we formulated our primary hypothesis that the AGm is necessary for online sensory-motor integration during skilled walking, where precise paw placement depends on moment-to-moment somatosensory feedback and attentional control.

Motor cortex infarction involving both the lateral agranular cortex (AGl) and AGm increases contralateral stepping errors in the ladder rung walking task, a skilled walking paradigm that assays precise paw placement and draws on multisensory inputs [11]. However, because these lesions span both areas, the independent contribution of the AGm remains unresolved. To directly test our primary hypothesis, we used the ladder rung walking task as an operational assay of sensory-motor integration and examined whether focal AGm-restricted lesions impair paw placement accuracy.

In addition to this primary aim, the AGm exhibits rostrocaudally organized connectivity consistent with potential functional heterogeneity [12,13]. Therefore, as a secondary exploratory aim, we induced focal photothrombotic infarctions at five rostrocaudal levels (+2.0, +1.5, +1.0, +0.5 mm anterior to bregma) and compared behavioral performance across these sites. This article was previously posted on the medRxiv preprint server on November 24, 2023.

## Materials and methods

Animals

Nine-week-old male C57BL/6J mice (Japan SLC Co., Shizuoka, Japan) were individually housed in cages under controlled conditions (12-h light/dark cycle, 23 ± 1ºC) with food and water ad libitum. To evaluate sensory-motor integration in the ladder rung walking task after cerebral infarction, mice were divided into five groups: the control group (n = 11) and four AGm-lesion groups-AGm2.0 (n = 15), AGm1.5 (n = 10), AGm1.0 (n = 8) and AGm0.5 (n = 9)-where the label denotes the anteroposterior coordinate (in mm) of the lesion center relative to bregma (i.e., +2.0, +1.5, +1.0, and +0.5 mm, respectively). All experiments were conducted in accordance with the “Guidelines for the Proper Conduct of Animal Experiments” established by the Science Council of Japan (2006) and were approved by the Animal Care and Use Committee of Ibaraki Prefectural University of Health Sciences (approval number 2022-14). This study is reported in accordance with the ARRIVE guidelines.

Photothrombotic infarction in the AGm

Focal photothrombotic infarction in the AGm was induced as previously described with minor adaptations to target multiple anteroposterior (AP) levels [14-18]. Briefly, anesthesia was induced with 5.0% sevoflurane and maintained with 2.0% sevoflurane; mice were placed in a stereotaxic apparatus, the skull was exposed and kept moist with saline. At 5 min after intraperitoneal injection of 1% Rose Bengal (100 mg/kg), a green light (532 nm) delivered via an optical fiber (diameter, 0.4 mm; output power, 11 mW; Thorlabs, Newton, NJ, USA) was applied for 15 min over the right AGm. For group-specific targeting, the optic fiber was positioned at one of the following AP coordinates relative to bregma: +2.0, +1.5, +1.0, or +0.5 mm, at 0.6 mm lateral to the midline in the right hemisphere. Controls underwent identical procedures, except that illumination with a light-emitting diode preceded Rose Bengal administration, preventing photochemical activation and infarct formation.

Ladder-rung walking task

**Figure 1:**
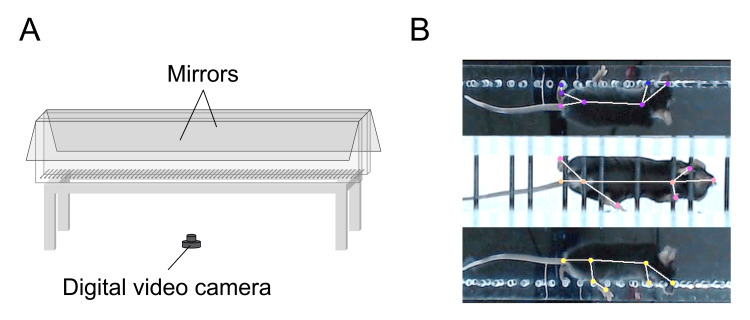
Experimental set-up of the ladder rung walking task (A) Schematic diagram of the experimental apparatus. A single camera placed below the ladder captured both bottom and lateral views via mirrors. (B) Example of a misstep on the ladder and markers used for postural analysis.

The ladder rung walking task was used to assess sensory-motor integration, as reflected by missteps [19] (Figure 1). The horizontal ladder apparatus (100 cm long, 3.2 cm wide, 56 cm high) and the recording system were adapted from a previous study (Figure 1A) [11]. Two conditions were tested: a regular pattern with alternating 1-cm spacing, and an irregular pattern with randomly spaced rungs ranging from 1 to 2 cm. In the irregular condition, no three consecutive rungs were spaced at 1 cm. Ten unique irregular patterns were generated in advance. For each mouse, one previously unused pattern was randomly assigned to each session (without replacement) and used for all irregular trials in that session, so no pattern was reused across the seven sessions for the same mouse. All mice were transferred to the testing room at least 1 h before each behavioral test to acclimate. All behavioral testing was conducted during the light phase in a quiet room with minimal external noise or disturbance.

Training began three days before the induction of focal photothrombotic infarction. Mice were trained for two consecutive days, performing five trials per day under the regular rung condition. Two days after completion of training, a pretest was conducted, immediately followed by induction of photothrombotic infarction. From day 2 to day 7 after infarction, posttests were conducted once daily for a total of six sessions. In both the pretest and each posttest session, mice performed six trials (three regular and three irregular).

Mouse movements were recorded simultaneously from three directions (left, right, and bottom) using two mirrors and a digital video camera (C922; Logitech, Lausanne, Switzerland) (Figure 1A). Videos (1920 × 1080 pixels) were cropped to 300 × 900 pixels and analyzed using DeepLabCut^TM^ (v2.2.0.4), a markerless pose-detection machine learning Python package [20]. A misstep was defined as the toe tip dropping 3 mm below rung height, and the error rate was calculated as errors/total steps (Figure 1B). We trained the neural network for 406,690 iterations on 172 labeled frames, indicating the positions of all four limbs, the nose, and the tail across three views. Limb missteps were detected using a custom Python program.

Nissl staining and slice analysis

Nissl staining and lesion-size quantification were performed as previously described [17,18]. Briefly, after the final behavioral session, brains were processed and 100-μm coronal sections were collected at 100-μm intervals and stained with 0.1% cresyl violet acetate (Sigma-Aldrich Co., LLC, Tokyo, Japan). Digital micrographs were acquired using a light microscope, and lesion borders were manually contoured on the images using Image J software (version 1.53; National Institutes of Health, Bethesda, MD, USA). For each section, the lesion area was defined as cortical tissue showing Nissl pallor relative to the surrounding cortex and was measured in mm². Lesion volume was then obtained by summing (area × section thickness, 0.1 mm) across all sections containing the lesion. In addition, the centroid of the binary lesion mask was computed for each section; anteroposterior centroid coordinates (relative to bregma) were extracted for subsequent analyses. Image processing was performed in ImageJ and numerical calculations in Matlab 2022a (MathWorks, Natick, MA, USA). For visualization of spatial consistency, binarized lesion masks from individual animals were registered to corresponding stereotaxic atlas levels and stacked in Matlab to generate two-dimensional lesion overlap maps plotted along the anterior-posterior and medial-lateral axes.

Statistical analysis

All analyses were conducted with SPSS software, version 23.0 for Windows (IBM Corp., Armonk, NY, USA). Data are presented as the mean ± standard error of the mean. Group differences in infarct volume were tested with one-way analysis of variance (ANOVA). Ladder rung error rates were analyzed with two-way repeated-measures ANOVAs run separately for forelimb/hindlimb and for regular/irregular conditions, with within-subject factors Side (left, right) and Session (7 levels: pretest and postoperative days 2-7). In all ANOVA models, whenever a relevant effect was significant, post hoc pairwise comparisons used Bonferroni adjustment. Statistical significance was considered at p < 0.05. Effect sizes were calculated for all major analyses, with partial η² used for ANOVA effects and Cohen’s dz for paired comparisons.

## Results

Photothrombotic infarction in AGm2.0, 1.5, 1.0, and 0.5

**Figure 2:**
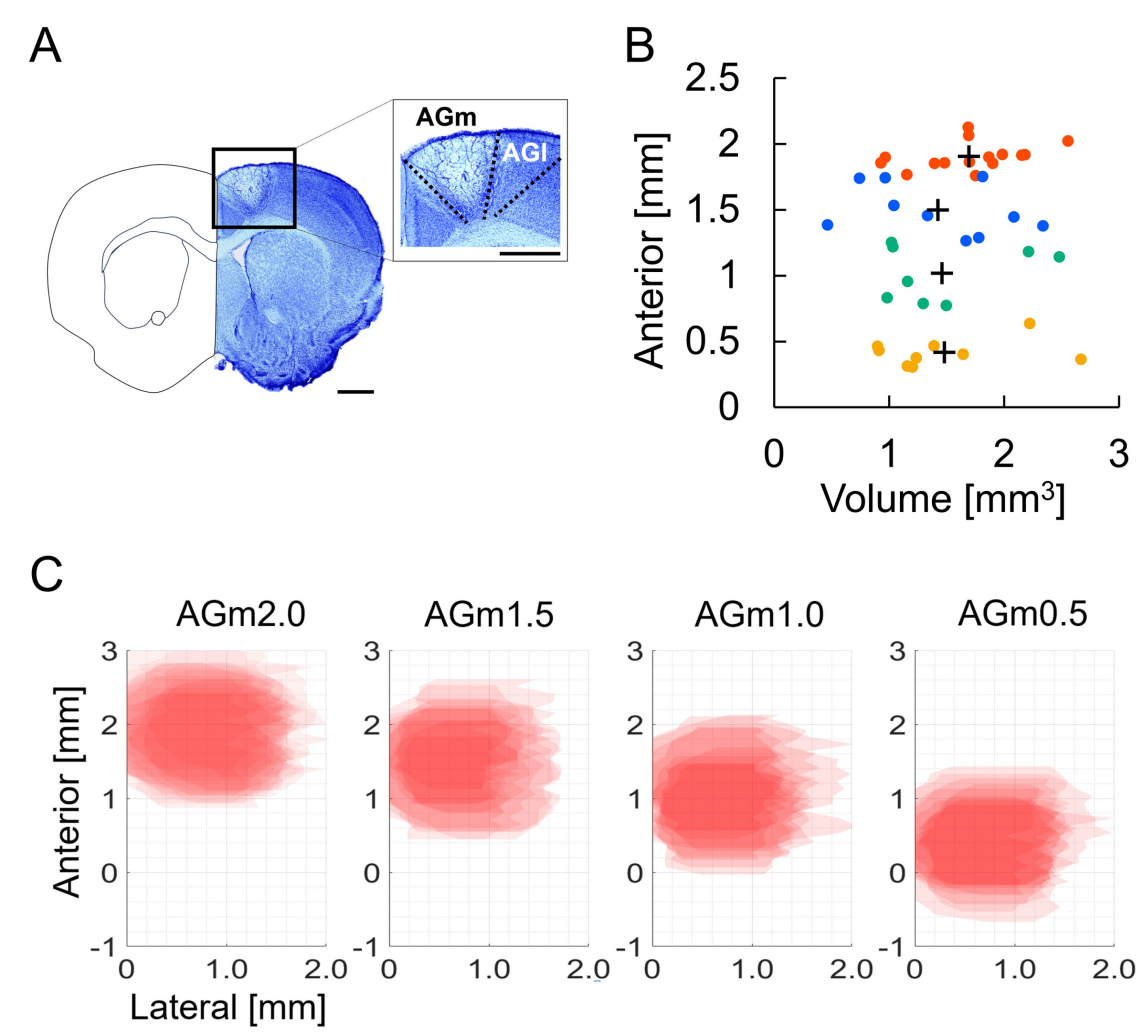
Photothrombotic infarction in AGm (A) Representative Nissl-stained coronal brain section. Scale bar, 1 mm. (B) Scatterplot of the anterior–posterior (A–P) lesion centroid (y-axis, relative to bregma) versus infarct volume (x-axis) on postoperative day (POD) 7. Points denote individual mice; “+” indicates the group mean (mean A–P centroid and mean volume) for each group. Orange: AGm2.0 (n = 15); blue: AGm1.5 (n = 10); green: AGm1.0 (n = 8); yellow: AGm0.5 (n = 9). (C) Lesion overlap maps across animals. Lesion boundaries from each mouse were traced and registered to corresponding atlas levels. Colormaps indicate the number of overlapping lesions per pixel, with darker colors representing greater overlap.

Figure 2A shows a representative example of Nissl-stained sections. Infarction was confirmed in the AGm of all animals. The centroid coordinates of the lesion area along the anterior-posterior axis and the lesion volumes on postoperative day (POD) 7 in all groups are shown in Figure 2B. There was no difference in infarct volume among groups (F(3, 41) = 0.629, p = 0.601, partial η² = 0.047; Figure 2B). To visualize spatial consistency across animals, lesion masks were traced and registered to stereotaxic atlas levels, and colormaps were generated to indicate the degree of lesion overlap across subjects (darker colors represent greater overlap; Figure 2C). The overlap maps confirmed that infarcts consistently encompassed the AGm in all animals.

No deficit in ladder-rung walking performance after AGm infarction

**Figure 3:**
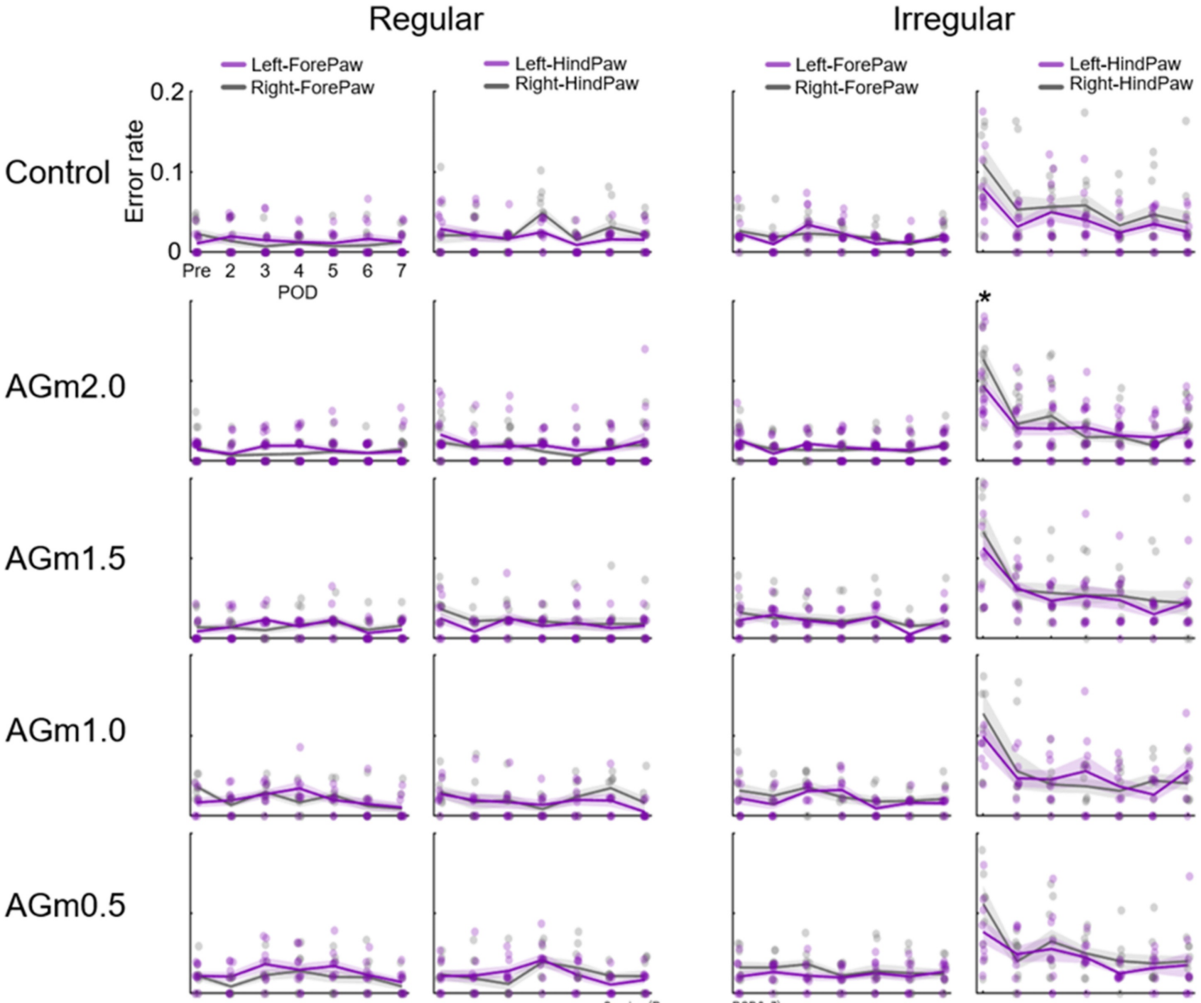
Stepping error rates during the ladder rung walking task Time course of foot-fault (error) rates for the left (contralesional) and right (ipsilesional) forelimbs and hindlimbs under regular and irregular conditions. For each group and condition, error rates (errors/steps) were calculated separately for forelimbs and hindlimbs. Lines represent group mean error rates, shaded areas indicate the SEM, and semi-transparent dots represent data from individual animals, with purple indicating left limbs and gray indicating right limbs. “Pre” denotes the baseline error rate obtained before infarct induction. POD indicates postoperative day, i.e., the number of days after infarction. Asterisks denote significant left-right differences within a session (Bonferroni-corrected paired comparisons following two-way repeated-measures ANOVA; p < 0.05).

To quantify performance, we computed foot-fault rates (errors/steps) on the ladder rung walking task across seven sessions (pretest and postoperative days 2-7). Except for the AGm2.0 group, two-way repeated-measures ANOVAs with factors Side (left vs right) and Session (7 levels), run separately for forelimb/hindlimb and for regular/irregular conditions, revealed no significant Side × Session interactions (all p > 0.05, partial η² range = 0.03-0.21). In the AGm2.0 group (hindlimb, irregular condition), the interaction between Side and Session was statistically significant (F(6, 84) = 3.073, p = 0.009, partial η² = 0.18; Figure 3). Bonferroni-corrected post hoc paired comparisons (right vs left within each session) showed that the right hindlimb had a higher error rate than the left at pretest (p < 0.05, Cohen’s dz = 0.64), whereas no side differences were detected on any postoperative day (p > 0.05, Cohen’s dz = 0.04-0.53).

## Discussion

This study investigated whether lesioning the AGm in mice induces deficits in sensory-motor integration as assessed with the ladder rung walking task. Across the postoperative sessions (days 2-7), foot-fault rates did not increase for either the forelimbs or the hindlimbs under regular and irregular rung patterns. Within the sensitivity of this assay and under the present conditions, we detected no impairment in ladder rung performance after AGm infarction. In the AGm2.0 group (hindlimb, irregular condition), a right-greater-than-left difference was observed at pretest only and did not persist after surgery. The absence of postoperative lateralization argues against a lesion-induced lateralized deficit.

Given that the AGm has corticospinal projections and reciprocal connections with S1 neurons, it has been proposed to integrate sensory information and motor commands for limb control [7]. Thus, damage to the AGm might be expected to impair motor performance involving sensory-motor integration. However, in the context of the ladder rung task, our results indicate that the AGm is not critically required for precise paw placement guided by visual and somatosensory inputs.

In contrast to our AGm-restricted lesions, motor cortex infarcts that encompass both the AGl and the AGm produce increased contralateral stepping errors on the ladder rung task [11]. Intracortical microstimulation (ICMS) mapping delineates two forelimb fields: a large caudal forelimb area (CFA) that largely overlaps the AGl, and a smaller rostral forelimb area (RFA) that partially overlaps the rostral AGm [21]. Within this framework, our finding that AGm-only infarction left ladder rung performance intact indicates that motor execution is primarily supported by AGl/CFA. By contrast, combined AGl+AGm infarction disrupts stepping, implying that the AGm plays a modulatory, rather than essential, role in sensory-motor control.

Moreover, the rostral AGm receives greater somatosensory inputs, whereas its central and caudal parts receive more visual inputs [13,22]. In our previous study, recovery from unilateral spatial neglect (USN) was slower with more rostral AGm lesions, suggesting location-dependent functional differences within the AGm [17,18]. By contrast, in this present study, we observed no site-dependent differences in postoperative ladder rung performance across five rostrocaudal lesion sites, indicating no detectable site effect on limb motor execution under the present task conditions. Taken together, these observations suggest that rostrocaudal specialization within the AGm is more salient for attentional processes than for motor execution in this task context. This interpretation is consistent with previous evidence linking the AGm to spatial attention [1-3] and with our USN mouse model induced by AGm lesions [17,18]. Overall, our results support a model in which the AGm contributes to higher-order control, particularly spatial attentional control, rather than to the basic sensorimotor processes required for stepping in this task. [4,23,24].

Alternative explanations for the negative results should also be considered. One possibility is that compensatory neural plasticity in adjacent cortical areas, such as the AGl or S1, may have rapidly restored functional output after focal AGm infarction, as shown in previous studies demonstrating functional reorganization in peri-lesional or neighboring cortical regions following motor cortex injury [25]. The adaptive organization of motor circuits may also have masked subtle behavioral deficits, allowing other premotor or somatosensory regions to maintain stepping accuracy [25-27]. From a translational perspective, elucidating why AGm lesions do not produce overt motor deficits may help clarify how compensatory mechanisms support functional recovery after stroke. In humans, spared premotor or parietal regions may play analogous roles in restoring sensory-motor integration and attentional control following cortical damage [28,29].

This study has several limitations. First, infarcts were induced only in the AGm of young adult male mice; therefore, the findings may not generalize to females or aged animals. Second, although the ladder rung walking task is a sensitive assay of sensory-motor integration, the absence of deficits in this task raises the possibility that AGm lesions may selectively affect functions more dependent on attentional control rather than basic motor execution. Tasks that place higher demands on attention, such as visually guided reaching or divided-attention paradigms, may therefore reveal AGm-dependent impairments even when basic stepping accuracy remains intact [30]. Finally, although missteps were quantified using automated tracking, subtle gait alterations not detected by this metric may have been overlooked. Accordingly, future studies should include female and aged cohorts and employ multiple behavioral assays beyond the ladder rung task.

## Conclusions

In this study, we examined the role of the medial agranular cortex (AGm) in sensory-motor integration by assessing the impact of AGm lesions on motor performance in the ladder rung walking task. Despite its corticospinal projections and connectivity with primary sensory cortices, AGm lesions did not increase foot-fault rates on the ladder rung task across five rostrocaudal lesion sites within the sensitivity of this assay. These findings indicate that the AGm is not required for accurate limb placement under this task. Instead, in concert with prior evidence, our results support the view that the AGm contributes more critically to higher-order functions such as spatial attention, rather than to direct motor execution. By dissociating the AGm’s role from primary motor control, this study helps clarify how cortical networks for movement and attention are functionally segregated yet interact to support adaptive behavior. Understanding this division of labor between motor and attentional systems may provide a framework for interpreting recovery and compensation following cortical injury.
